# A rapid method to reduce drug interferences for antibody measurements in pegunigalsidase alfa-treated patients with Fabry disease

**DOI:** 10.3389/fimmu.2025.1724835

**Published:** 2026-01-13

**Authors:** Malte Lenders, Elisa Rudolph, Michael Rudnicki, Markus Cybulla, Eva Brand

**Affiliations:** 1 Internal Medicine D (Nephrology, Hypertension and Rheumatology), and Interdisciplinary Fabry Center (IFAZ), University Hospital Muenster, Muenster, Germany; 2 Department of Internal Medicine IV – Nephrology and Hypertension, Medical University Innsbruck, Innsbruck, Austria; 3 Department of Nephrology and Rheumatology, FGM, Center of Internal Medicine, Müllheim, Germany

**Keywords:** anti-drug antibodies, drug interferences, enzyme replacement therapy, Fabry disease, pharmacokinetics

## Abstract

**Aim:**

Neutralizing anti-drug antibodies (ADA) limit therapy efficacies significantly. Pegunigalsidase alfa is a newly approved drug for the treatment of Fabry disease. The increased plasma half-life due to PEGylation interferes with ADA measurements including ELISAs and serum-mediated inhibition assays. We developed a rapid protocol eliminating pegunigalsidase alfa from blood samples, without interfering downstream applications.

**Methods:**

A rapid protocol based on alkaline-pretreatment followed by acid-based neutralization of agalsidase-spiked control sera was established. Results were confirmed using serum samples from patients with and without neutralizing ADAs drawn during infusions. Repeated ADA measurements including serum-mediated inhibition assays and ELISA-based immunoglobulin isotyping (IgG, IgA, IgM) were performed with sera from 17 patients receiving pegunigalsidase alfa.

**Results:**

Alkaline pretreatment with NaOH was sufficient to eliminate up to 1 µg/ml agalsidase alfa or pegunigalsidase alfa in control sera. AGAL activities in sera drawn during infusions were completely suppressed without interfering subsequent serum-mediated inhibition assays. Based on this method, in one patient a *de novo* formation of ADAs against pegunigalsidase alfa was identified. Immunoglobulin isotyping showed mainly IgM antibodies towards pegunigalsidase alfa, recognizing PEG moieties and amino acids in this patient. Although his ADAs had a low inhibitory capacity, Western blot analyses demonstrated that the reduced pharmacokinetics might be linked to leucocyte-mediated enzyme elimination. A second patient with pre-existing ADAs before pegunigalsidase alfa-initiation showed a massive induction of anti-PEG antibodies with inhibitory function.

**Conclusion:**

We present a rapid alkaline-treatment based method to overcome drug interferences to measure at least free antibodies in patients treated with pegunigalsidase alfa.

## 1 Introduction

Fabry disease (FD) is a lysosomal storage disease, caused by a deficiency of the enzyme α-galactosidase A (AGAL). The enzymatic deficiency results in a cellular accumulation of the main substrate globotriaosylceramide (Gb_3_), leading to a multisystemic disease with manifestations such as heart failure, cardiac arrhythmia, stroke or transient ischemic attacks, as well as end-stage renal disease (1).

Since 2001 FD is treatable by enzyme replacement therapy (ERT) including agalsidase alfa (0.2 mg/kg body weight (BW) every other week (e.o.w.); Shire/Takeda), or agalsidase beta (1.0 mg/kg BW e.o.w.; Sanofi-Genzyme) and since 2023 by pegunigalsidase alfa (1.0 mg/kg BW e.o.w.; Chiesi Therapeutics) intravenously (2–5). Treatment with all three compounds showed beneficial effects on disease manifestation and progression in affected patients over time. Due to the X-linked inheritance of FD, classical male patients with deleterious GLA mutations who are unable to form any native AGAL enzyme are cross-reactive immunologic material negative. Similar to patients with other ERT treatable diseases (6), they are at a high risk of forming neutralizing anti-drug antibodies (ADAs) against agalsidase alfa, agalsidase beta, and pegunigalsidase alfa, which significantly decrease the therapeutic efficacy of ERT (7–11). In addition, the overall frequency for anti-PEG antibodies is reported as ~40% (12). Thus, all patients treated with pegunigalsidase alfa might already have pre-existing or develop *de novo* anti-PEG antibodies, which can also affect the therapy efficacy significantly (13). In a previous study we demonstrated that pre-existing neutralizing ADAs reduce the pharmacokinetic profile of pegunigalsidase alfa (14). These data in addition to the clinical phase I–III studies (4, 5, 15, 16) highlight the importance of antibody measurements in patients treated with the new drug. However, the long plasma half-life of pegunigalsidase alfa interferes with the detection of free antibodies against the amino acid sequence and PEG moieties, leading to false low antibody titers (17, 18). Furthermore, the remaining active enzyme interferes with serum-mediated inhibition assays (7, 8), which are most suitable to detect neutralizing ADAs in patients at risk.

In the current study, we provide a simple and rapid protocol to eliminate and suppress functional pegunigalsidase alfa from serum samples, without interfering downstream applications including serum-mediated inhibition assays and ELISA-based antibody measurements and immunoglobulin isotyping. After successful establishment, the protocol was evaluated by antibody measurements in sera of 17 patients newly treated with pegunigalsidase alfa.

## 2 Materials and methods

### 2.1 Patients

All investigations were performed after approval by the Medical Association of Westphalian-Lippe and the Ethics Committee of the Medical Faculty of the University of Muenster (project no. 2011-347-f, date of report: 7 July 2011) and in accordance with the Declaration of Helsinki. Written informed consent was obtained from all included patients for analysis and publication. In total, 17 patients receiving pegunigalsidase alfa were recruited between June 2023 and April 2025. Blood drawings were performed immediately before and 15–30 minutes after infusions from the opposite arm.

### 2.2 α−Galactosidase A activity measurements in serum samples

AGAL activity measurements in serum samples were performed as previously described (14). In short, AGAL activities in 5 μl of serial dilutions of sera (diluted with fetal calf serum (FCS)) were measured using the 4-methylumbelliferyl α-D-galactopyranoside (4-MUG) method in 96-wells plates. On each plate, serial dilutions of either pegunigalsidase alfa or agalsidase beta (starting at 1.25 ng AGAL) were incubated with the substrate 4-MUG and the resulting fluorescence (AGAL activity) was determined. Following this concept, the measured activity (RFU) correlates with the amount of enzyme (ng), allowing the expression of the measured AGAL activities in ng AGAL per ml serum. Pegunigalsidase alfa was used for a standard curve to express enzymatic AGAL activities in pegunigalsidase alfa-treated patients. The mean recovery rate of pegunigalsidase alfa in our assays was 99.7 ± 6.9%.

### 2.3 AGAL detection in serum samples and blood mononuclear cells

To detect pegunigalsidase alfa in serum samples, 20 µg total protein from sera drawn before or after infusions were separated on a 10% SDS gel. Subsequently, the samples were blotted onto a PVDF membrane and blocked overnight in 5% semi-skimmed milk powder (Roth, Karlsruhe, Germany) in Tris-buffered saline supplemented with 0.1% Tween 20 (TBST; AppliChem, Darmstadt, Germany). Anti-AGAL (ab16834, Abcam, 1:5,000) was used to detect pegunigalsidase alfa and visualized with a secondary horseradish peroxidase-conjugated goat ant-rabbit antibody (Merck Millipore, Burlington, Massachusetts, USA, 12-348, 1:10,000).

To detect pegunigalsidase alfa (and endogenous AGAL) in PBMCs, cells were isolated from 7.5 ml EDTA-stabilized blood samples drawn before and after infusions. A total of 15 to 20 µg whole protein was separated on a 10% SDS gel and blotted onto a PVDF membrane.

### 2.4 Alkaline-based α-galactosidase A inactivation and activity measurements

**Figure 1:**
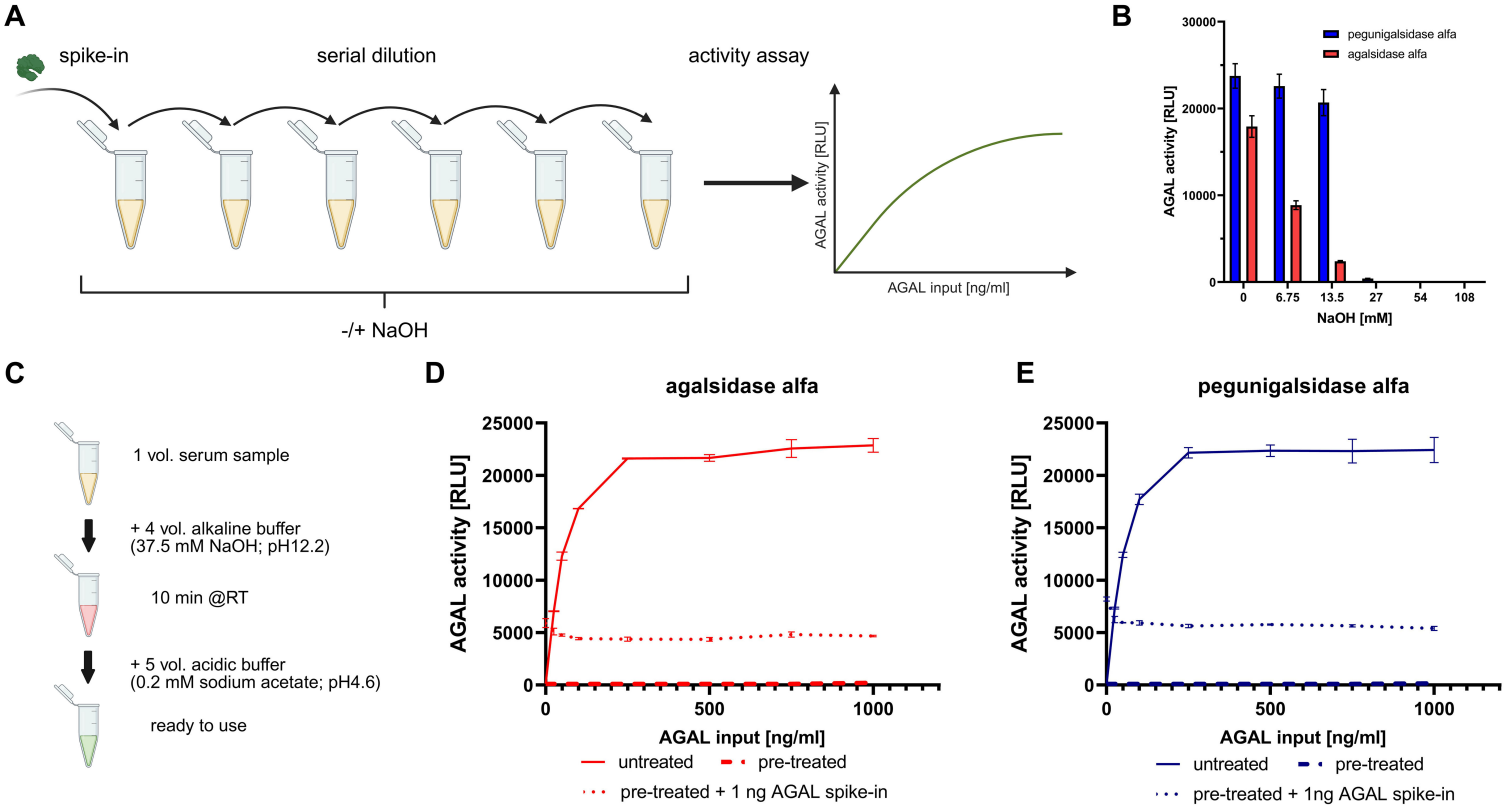
Overview of the procedure for establishing a protocol to eliminate pegunigalsidase alfa-mediated drug interference in antibody measurements. **(A)** Basic procedure and dilution series with AGAL-spiked serum. **(B)** NaOH concentration-dependent inactivation of 10 ng agalsidase alfa (red bars) or pegunigalsidase alfa (blue bars), demonstrating that an end concentration higher than 27 mM NaOH was sufficient to eliminate activities of both enzymes. **(C)** Overview of the complete alkaline-based protocol followed by neutralization for subsequent serum-mediated inhibition assays. Alkaline pretreatment-mediated inactivation with an end concentration of 30 mM NaOH of up to 1000 ng/ml agalsidase alfa (red) **(D)** or pegunigalsidase alfa (blue) **(E)** demonstrates the successful sustained enzyme inactivation without interfering with subsequent spiked-in 1 ng AGAL. These data also indicate that the pretreatment with NaOH leads to a persistent inactivation of the enzyme, which cannot be revoked by neutralization with acidic buffer.

After assessing the final sodium hydroxide (NaOH) concentration, one volume of AGAL-spiked-in FCS samples or human sera were incubated for 10 minutes at room temperature with 4 volumes of the dissociation buffer (pH12.2, treated samples) or 0.9% sodium chloride (NaCl; untreated samples). For subsequent neutralization, five volumes of 0.2 M sodium acetate (NaAc) were added (**Figure 1**). Samples were used directly or stored at 4°C for up to one week. AGAL activities were measured as previously described (8), using an end-concentration of 100 mM NaAc, and 4-methylumbelliferyl-α-D-galactopyranoside (4-MUG; Biosynth, Staad, Switzerland) as substrate. After incubation for 1 hour at 37°C, the enzymatic reaction was stopped by adding one volume of 2 M sodium carbonate (Na_2_CO_3_). The fluorescence intensity was measured at 365/465 nm wave length using a microplate reader.

### 2.5 Serum-mediated AGAL inhibition assays

Serum-mediated inhibition assays were performed as previously described (7, 8), using either alkaline-pretreated or untreated sera. Inhibition was tested against agalsidase alfa and pegunigalsidase alfa and compared to a negative control (FCS).

### 2.6 ELISA-based antibody measurements

ELISAs were performed as recently described (14). In detail, wells of 96-well Maxisorp ELISA plates were precoated with either 100 ng agalsidase alfa or pegunigalsidase alfa or 200 ng PEG5000-BSA (NANOCS, PG5k-BS, New York, NY, USA) accordingly. These different approaches allowed discrimination between AGAL- and PEG-specific antibodies. Coated plates were subsequently blocked with 5% BSA in phosphate-buffered saline (PBS), and incubated with serial dilutions of patients’ sera alkaline-pretreated sera, starting with 1 µl serum input. After 2 hours, wells were washed five times with 0.1% Chaps/ PBS. To detect IgG isotype-specific antibodies mouse anti-human IgG isotype 1 [ab99774], mouse anti-human IgG isotype 2 [ab99779]; rabbit anti-human IgG isotype 3 [ab86253]; mouse anti-human IgG isotype 4 [ab99823] were used. Working concentrations were 20 ng/ml. IgG3 detection required visualization with a HRP-coupled anti-rabbit antibody. Anti-human immunoglobulin M (IgM) (ab97205, Abcam; working concentration: 20 ng/ml), or anti-human immunoglobulin A (IgA) antibodies (ab97215, Abcam; working concentration: 20 ng/ml) were used for IgM and IgA detection, respectively. 50 μl 1-Step TMB-ELISA substrate solution (Thermo Fisher Scientific) was added to the wells, followed by 50 μl 2 M sulfuric acid to stop the reaction after 4–10 min. Absorption was measured at 450 nm. To exclude potential false negative results, we also tested our ELISA-based antibody measurements for unspecific bindings. In detail, 96 wells were coated with BSA (100 µg per well), and ELISAs were performed as described above to detect the different Igs, showing no significant signals due to unspecific antibody bindings (**Supplementary Figure 1**). A control ELISA with our patient-derived anti-AGAL reference antibody shows that the inhibitory IgG_4_ anti-AGAL antibodies have a significantly reduced affinity for the pretreated and neutralized pegunigalsidase alfa and therefore no longer recognize the enzyme (**Supplementary Figure 2**).

### 2.7 Statistical analyses

Measures were performed in triplets, only ELISAs were performed in duplicates. Categorical data are expressed as numbers, and relative frequencies as percentages. Cartoons were created with BioRender.com. GraphPad PRISM V8.4 software (GraphPad Software Inc, La Jolla, California) was used for appropriate statistical analyses and visualization.

## 3 Results

### 3.1 Establishment of a simple and rapid protocol for the removal of drug interferences for antibody measurements

**Figure 2:**
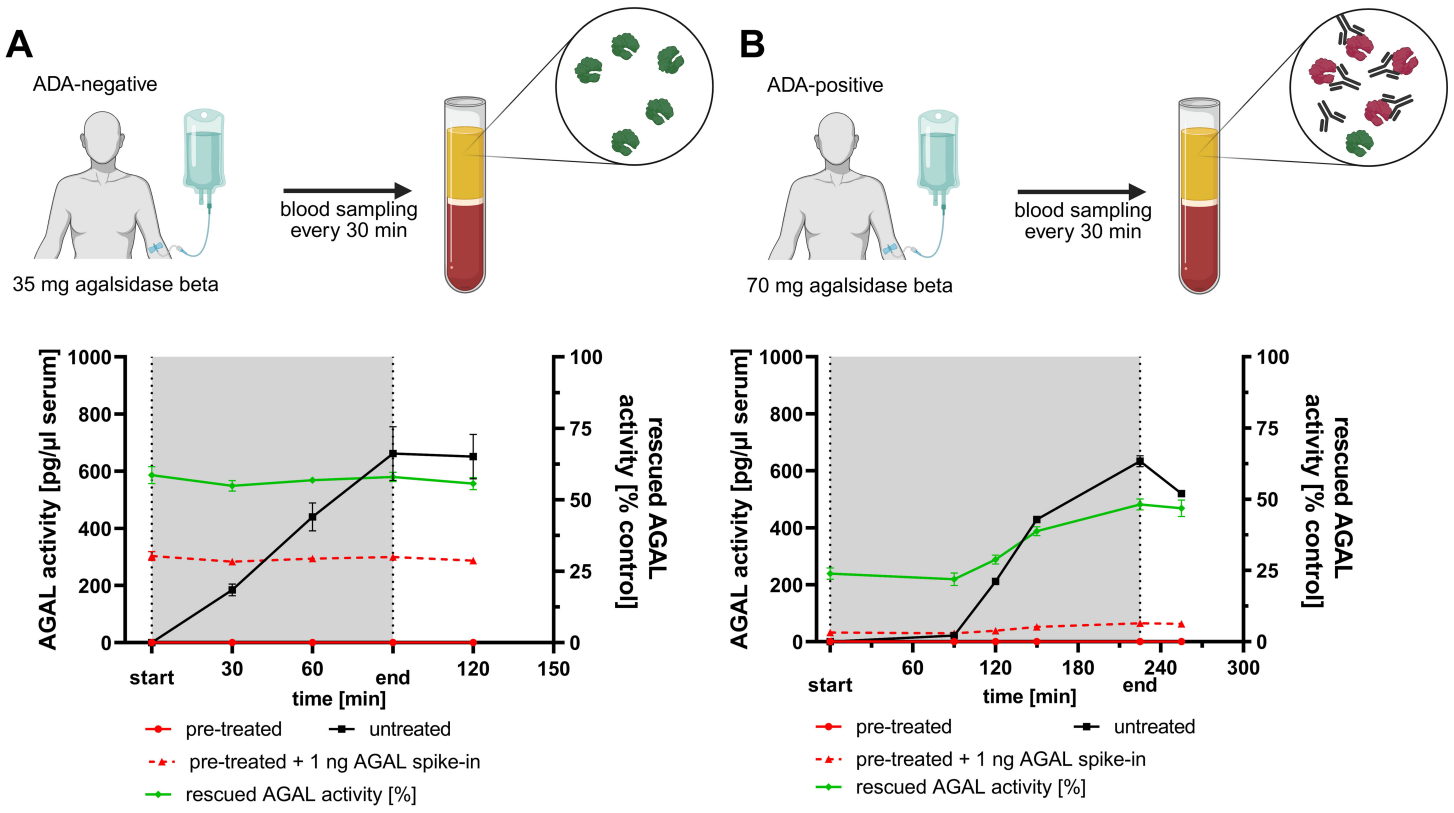
Transfer and validation of the protocol to human samples. **(A)** Male patient without anti-drug antibodies (ADA-negative) infused with a total of 35 mg agalsidase beta over 120 minutes. **(B)** Male patient with neutralizing ADAs (ADA-positive) infused with a total of 70 mg agalsidase beta over 240 minutes. Free ADAs in this patient were saturated after 90 minutes, visualized by an increase of the AGAL activity in the untreated serum samples (black line).

As recently discussed (14), the remaining circulating AGAL due to enzyme replacement therapy or gene therapy can interfere with assays for the detection and measurement of (neutralizing) ADAs. Thus, we aimed to establish a simple and rapid protocol, which is able to eliminate remaining AGAL in serum samples without affecting free ADAs in patients’ serum samples (**Supplementary Figure 3**). AGAL is a lysosomal enzyme with a catalytic optimum in acidic pH and thus prone to an alkaline pH. In a first step we used serial dilutions of AGAL spiked in FCS to elaborate the required concentration of alkaline sodium hydroxide (NaOH) in the samples to eliminate AGAL activities of agalsidase alfa and pegunigalsidase alfa (**Figure 1**). As expected, pegunigalsidase alfa was more stable and less sensitive to treatment with NaOH compared to agalsidase alfa (**Figure 1B**). However, an end concentration higher than 27 mM NaOH was sufficient to eliminate activities of both enzymes. Next, we analyzed the limits of the maximum enzyme quantity inactivated by NaOH and (importantly) if the pretreated samples can be used after neutralization with acidic buffer for subsequent inhibition assays in our chosen experimental setup (**Figure 1C**). An end concentration of 30 mM NaOH was capable to eliminate agalsidase alfa and pegunigalsidase alfa concentrations of 1000 ng/ml (**Figures 1D, E**). Furthermore, if the pretreated samples were neutralized with acidic NaAc buffer, a subsequent spike-in of 1 ng AGAL per sample resulted in appropriate measurable enzyme activities (**Figures 1D, E**). These data also indicate that the pretreatment with NaOH leads to a persistent inactivation of the enzyme, which cannot be revoked by neutralization with acidic buffer (**Figures 1D, E**). Next, we evaluated this experimental setup with human samples. For this purpose, serum samples from a patient negative for neutralizing ADAs and a patient positive for neutralizing ADAs were analyzed at regular intervals during infusion with agalsidase beta [35 mg and 70 mg, respectively] (**Figure 2**). Untreated samples showed increasing AGAL activities depending on the infusion duration. Importantly, the AGAL activities in the patient with neutralizing ADAs were detected delayed after 90 minutes of infusion, confirming that neutralizing ADAs inhibit the enzyme already during infusion (9, 19). A pretreatment of the serum samples with 30 mM NaOH was again able to suppress the infused enzyme completely (**Figure 2**). A spike-in with 1 ng AGAL of the pretreated samples from the ADA-negative patient led to appropriate AGAL activities, corresponding to >50% rescued AGAL activities (**Figure 2A**). In contrast, enzymatic AGAL activities of pretreated samples from the ADA-positive patient also spiked-in with 1 ng AGAL were severely decreased, indicating the presence of yet free and thus unbound ADAs (**Figure 2B**). Interestingly, after 90 minutes, a slight increase of spiked-in AGAL activities and thus increased rescued AGAL activities was detected (**Figure 2B**). These data indicate that the patient’s circulating ADAs are saturated by the infused enzyme and that the pretreatment of the samples might not dissolve existing ADA/AGAL-complexes (if the ADAs have a high affinity for their epitopes). However, this experiment confirms previous data, demonstrating that ADAs can be saturated during infusion by appropriate AGAL concentrations (9, 19). Thus, we conclude that the alkaline pretreatment of serum samples with subsequent neutralization will allow at least the determination of free and unbound neutralizing antibodies in patient’s sera, free of drug interferences mediated by either long-lasting ERT such as pegunigalsidase alfa or for future not (yet) approved gene therapy.

### 3.2 *In vivo* analysis of the pharmacokinetic profile of pegunigalsidase alfa after infusion

**Figure 3:**
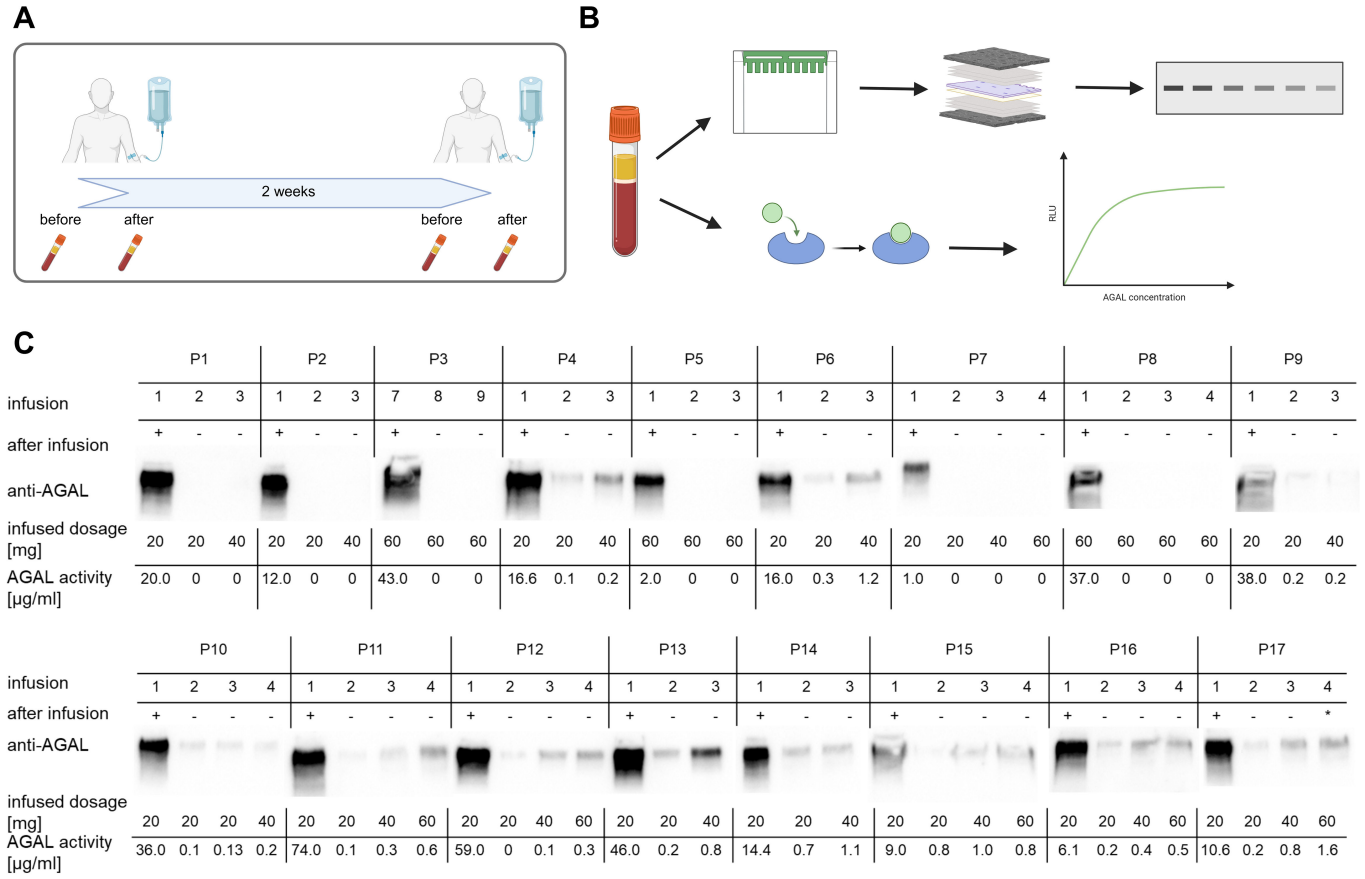
Overview of the blood samplings and subsequent α-galactosidase A detection. **(A)** Protocol for consecutive blood sampling, drawn before and after infusions. **(B)** Serum analyses included Western blotting and α-galactosidase A (AGAL) activity measures. **(C)** AGAL detection in serum samples of pegunigalsidase alfa-treated patients (n=17). Patients 2, 3, 5, 7, and 8 were positive for pre-existing neutralizing ADAs. In serum samples directly drawn before the next infusion, pegunigalsidase alfa was only detected (by Western blot and activity measurement) in patients without neutralizing antibodies. In patient 1 receiving migalastat before being switched to pegunigalsidase alfa and being negative for pre-existing anti-AGAL ADAs, pegunigalsidase alfa was absent before the next infusions, too.

**Figure 4:**
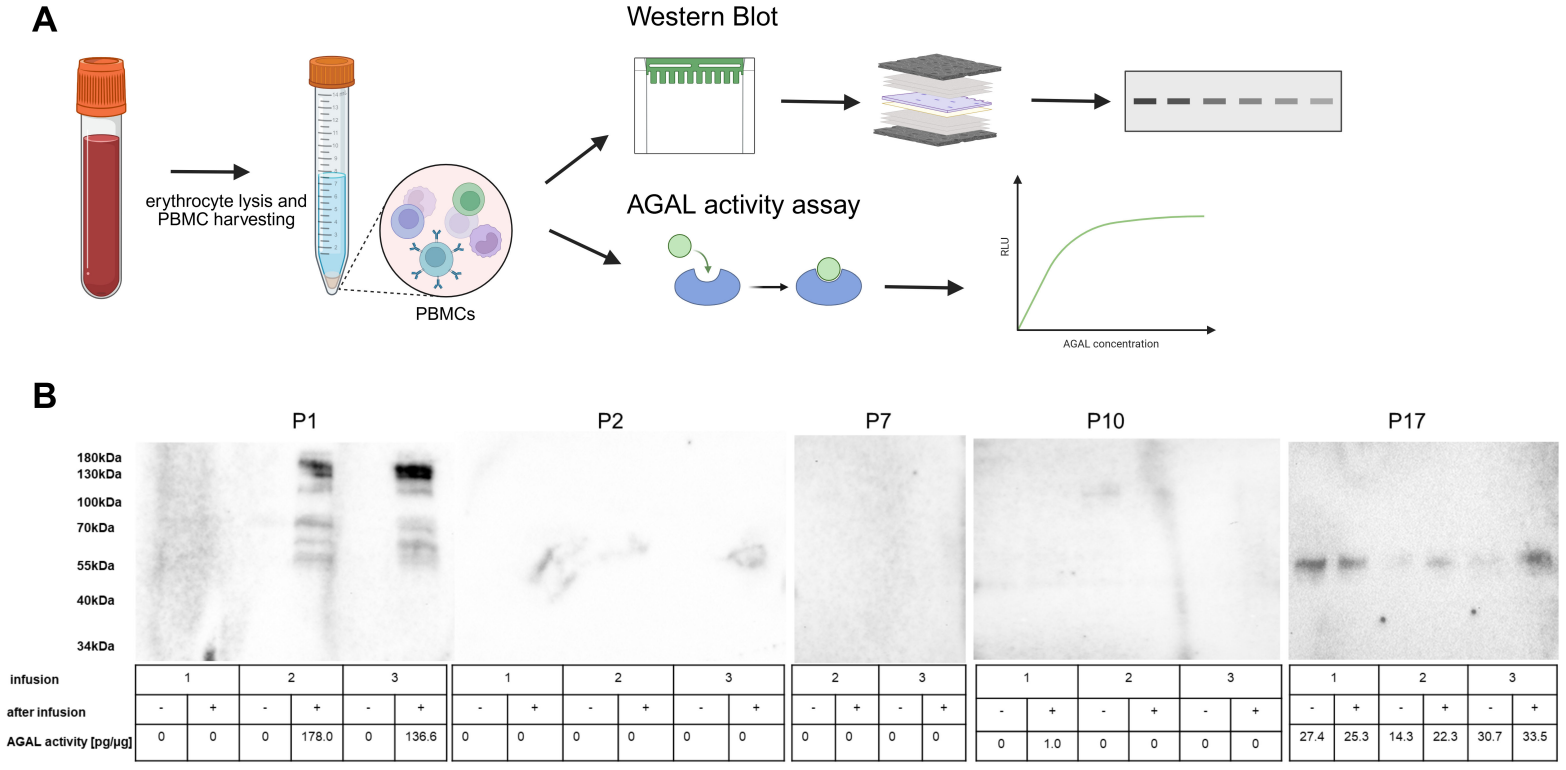
α-galactosidase A detection in available peripheral blood mononuclear cells. **(A)** Peripheral blood mononuclear cells (PBMCs) were isolated from EDTA-stabilized blood samples and analyzed by Western blot and enzyme activity measures for α-galactosidase A (AGAL) detection. **(B)** Outcomes for patients 1, 2, 7, 10, and 17 revealed pegunigalsidase alfa only in PBMCs of patient 1 after the second and third infusion. AGAL activity and signals in PBMCs from female patient 17 represent the endogenous AGAL. Only in PBMCs of patient 1 a distinct signal for full length and degraded pegunigalsidase alfa was detectable, while in PBMCs of patients with and without pre-existing anti-AGAL ADAs no pegunigalsidase alfa signals were detected.

Next, we aimed to transfer the pretreatment protocol to human samples to assess potential ADAs in patients newly treated with pegunigalsidase alfa (n=17, 6 females). In a first step, we analyzed serum samples consecutively drawn before and after infusions for the presence of pegunigalsidase alfa (**Table 1**, **Figure 3**). 11 patients were treated with either agalsidase alfa (n=3) or agalsidase beta (n=8) before being switched to pegunigalsidase alfa (**Table 1**). Five of the ERT-pretreated patients (all males) were positive for pre-existing neutralizing ADAs (patients: 2, 3, 5, 7, 8; **Table 1**). Three patients were previously treated with migalastat and 3 patients were naïve to any FD-specific therapy (**Table 1**). Western blot analyses and AGAL measurements in serum samples demonstrated pegunigalsidase alfa signals in all samples directly drawn after the infusions (**Supplementary Figure 4**, **Figure 3C**). In serum samples directly drawn before the next infusion, pegunigalsidase alfa was only detected (by Western blot and activity measurement) in patients without neutralizing antibodies (**Figure 3C**). Interestingly, in patient 1 who received migalastat before being switched to pegunigalsidase alfa and who was negative for pre-existing anti-AGAL ADAs, pegunigalsidase alfa was absent before the next infusions, too. To further elucidate the remainder of pegunigalsidase alfa, PBMCs also isolated before and after the infusions were analyzed for the presence of pegunigalsidase alfa in a subset of 5 patients, including patient 1 (**Figure 4**). Only in PBMCs of patient 1 a distinct signal for full length and degraded pegunigalsidase alfa was detectable, while in PBMCs of patients with and without pre-existing anti-AGAL ADAs no pegunigalsidase alfa signals were detected (**Figure 4**).

**Table 1:** Overview of the patients treated with pegunigalsidase alfa.

Number	Sex (f/m)	Age (years)	*GLA* variant	FD-specific pre-treatment	Pre-existing neutralizing ADAs	Pre-existing anti-PEG antibodies	Available consecutive visits after start	PBMCs available
1	m	61	p.I91T	migalastat	no	yes	3	yes
2	m	50	p.M42I	β	yes	no	3	yes
3	m	48	p.M1T	β	yes	yes	3	no
4	f	58	p.Y216X	β	no	no	3	no
5	m	54	c.718_719delAA	β	yes	yes	3	no
6	m	63	p.N215S	none	no	no	3	no
7	m	58	p.R220X	β	yes	no	4	no
8	m	37	nonsense	β	yes	yes	4	no
9	m	56	p.P259R	migalastat	no	yes	3	no
10	m	45	p.W349X	α	no	no	4	yes
11	f	62	c.762ins282bp	α	no	no	4	no
12	m	53	p.R49S	β	no	no	4	no
13	f	51	p.C63Y	none	no	yes	3	no
14	f	66	c.1021dupG	none	no	no	3	no
15	f	67	c.723dupT	β	no	yes	4	no
16	m	56	p.M290T	migalastat	no	yes	4	no
17	f	67	p.R220X	α	no	yes	4	yes
	6 /11	56 ± 8	9 nonsense		5 (29%)	9 (53%)		4/13

ADA, anti-drug antibody, GLA, α-galactosidase A, PEG, poly ethylene glycol, PBMC, peripheral blood mononuclear cells. f, females, m, male, α, agalsidase alfa, β, agalsidase beta.

### 3.3 Antibody detection using serum-mediated AGAL inhibition assays

**Figure 5:**
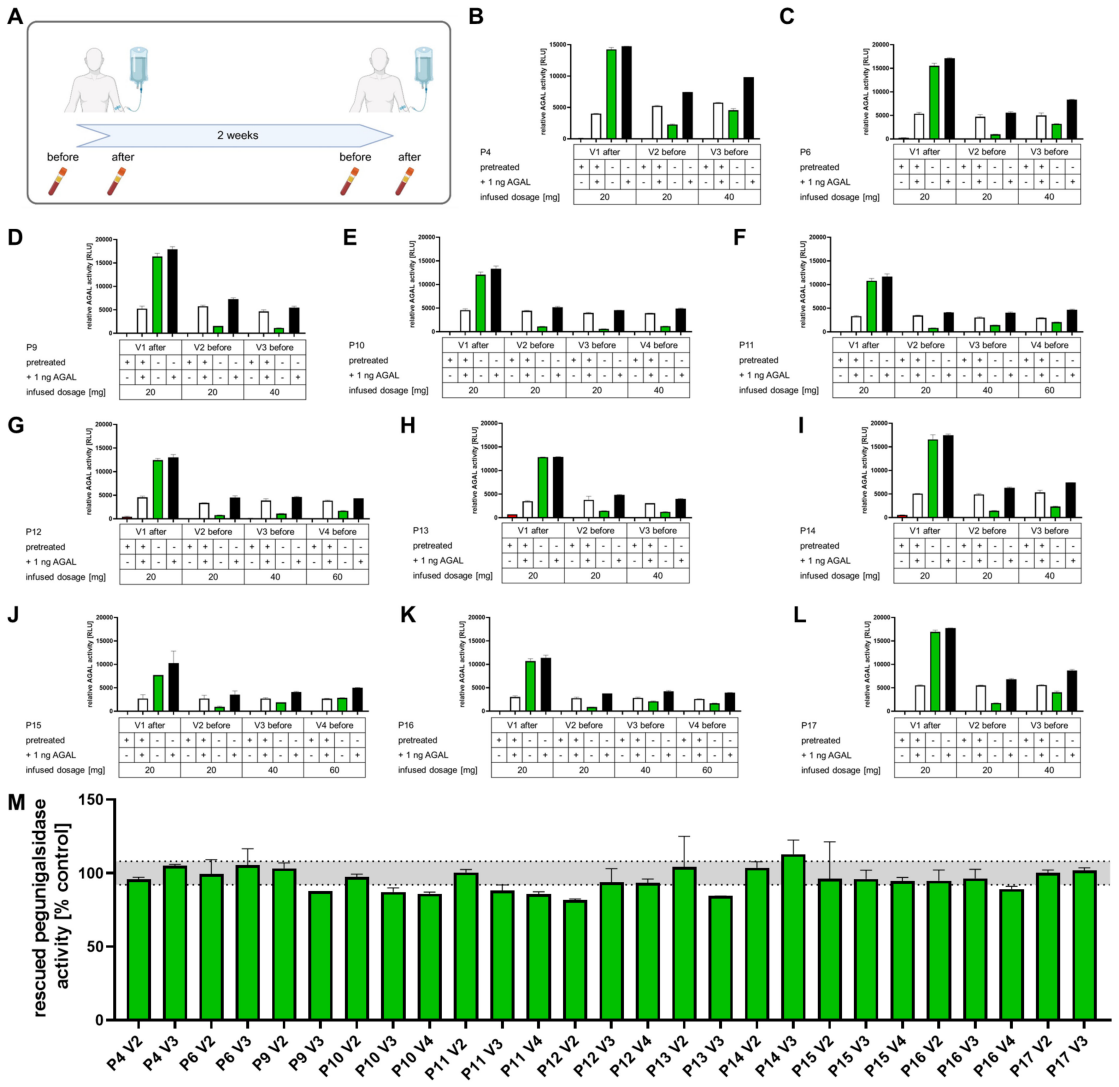
Serum-mediated inhibition assays with alkaline-pretreated serum samples from patients without neutralizing anti-drug antibodies. **(A)** Protocol for consecutive blood sampling. **(B-L)** Individual outcomes from serum-mediated inhibition assays against pegunigalsidase alfa. Four conditions were measured: alkaline-treated serum samples i) without and ii) with 1 ng spiked pegunigalsidase alfa, and untreated serum samples iii) without and iv) with 1 ng spiked pegunigalsidase alfa. Green bars: AGAL activities in untreated serum samples. Red bars: AGAL activities in alkaline pretreated serum samples. White bars: AGAL activities in alkaline pretreated samples spiked-in with 1 ng pegunigalsidase alfa. Black bars: AGAL activities in untreated samples spiked-in with 1 ng pegunigalsidase alfa. **(M)** Comparison of the rescued pegunigalsidase alfa activities from alkaline pretreated serum samples drawn directly before the next infusions.

**Figure 6:**
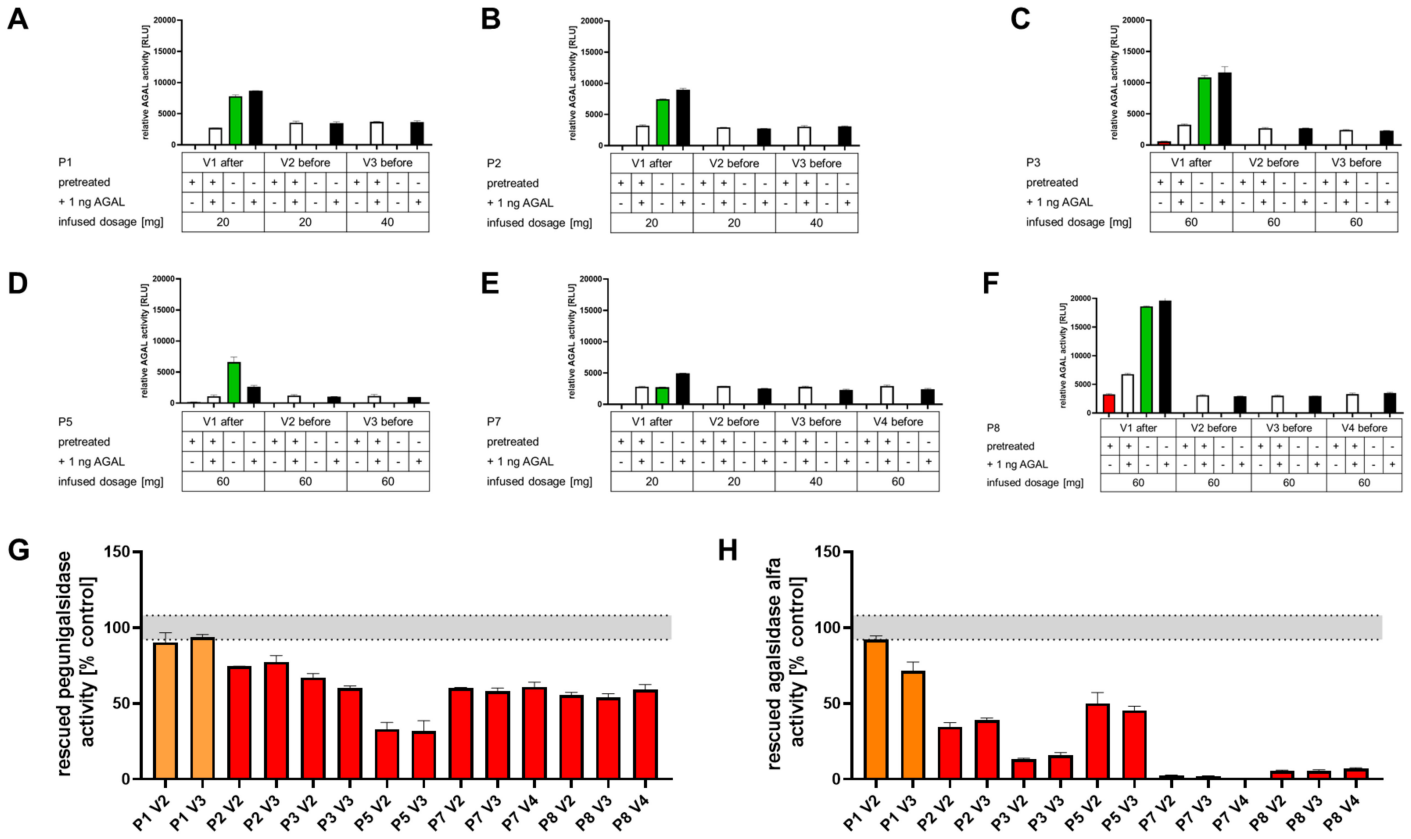
Serum-mediated inhibition assays with alkaline-pretreated serum samples from patients with neutralizing anti-drug antibodies. **(A-F)** Individual outcomes from serum-mediated inhibition assays against pegunigalsidase alfa. Four conditions were measured: alkaline-treated serum samples i) without and ii) with 1 ng spiked pegunigalsidase alfa, and untreated serum samples iii) without and iv) with 1 ng spiked pegunigalsidase alfa. Green bars: AGAL activities in untreated serum samples. Red bars: AGAL activities in alkaline pre-treated serum samples. White bars: AGAL activities in alkaline pretreated samples spiked-in with 1 ng pegunigalsidase alfa. Black bars: AGAL activities in untreated samples spiked-in with 1 ng pegunigalsidase alfa. **(G)** Comparison of the rescued pegunigalsidase alfa and **(H)** agalsidase alfa activities from alkaline pretreated serum samples drawn directly before the next infusions. The orange bars in **(G, H)** highlight rescued enzyme activities from serum samples of patient 1.

To analyze the functionality of the alkaline pretreatment-based protocol, we measured 4 conditions: alkaline-treated serum samples i) without and ii) with 1 ng spiked pegunigalsidase alfa and untreated serum samples iii) without and iv) with 1 ng spiked pegunigalsidase alfa (**Figures 5**, **6**). All untreated serum samples from patients without pre-existing neutralizing ADAs showed measurable AGAL activities, which could furthermore be increased by addition of 1 ng pegunigalsidase alfa (spike-ins). As expected, AGAL activities in serum samples drawn directly after the infusions (green bars) were higher than those drawn 2 weeks later before next infusions (**Figure 5**). Alkaline pretreatment of serum samples (red bars) led to absent measurable enzymatic AGAL activities in most samples (**Figure 6**). Only in some samples directly drawn after the infusions a residual AGAL activity was measured. By contrast, in serum samples drawn before the infusions, alkaline pre-treatment resulted in a full suppression of the remaining AGAL activities (**Figure 5**). A spike-in of 1 ng pegunigalsidase alfa of alkaline pretreated samples resulted again in measurable AGAL activities (white bars). Since these activities (white bars) roughly correspond to the spiked activities (black bars) minus the unspiked activities (green bars) of untreated samples, a proper function of this assay can be concluded. Next, we compared the alkaline pretreated samples spiked with 1 ng pegunigalsidase alfa to a control, similar to classic serum-mediated inhibition assays to assess the percentage of rescued AGAL activities (**Figure 5**). All serum samples from patients without preexisting ADAs showed a high percentage of rescued AGAL activity, confirming the absence of neutralizing ADAs (**Figure 5**).

Serum samples from patients with neutralizing ADAs showed a comparable picture. However, as expected, AGAL activities in untreated samples drawn before the next infusion were not detectable and the spike-in of 1 ng pegunigalsidase alfa resulted in lower AGAL activities (**Figure 6**). The comparison of residual AGAL activities from alkaline pretreated samples spiked with 1 ng pegunigalsidase alfa showed a notable reduction of rescued AGAL activities for nearly all samples expect those from patient 1 (**Figure 6**). Subsequent inhibition assays demonstrated only a slight inhibition for serum samples from this patient (**Figure 6**). The low inhibition in the serum of this patients might either point towards low titers of free circulating antibodies at the time-point of blood drawings or that nearly all potential epitopes were already recognized by the patient’s antibodies. To exclude the later one, we repeated the spike-in experiments with 1 ng agalsidase alfa instead of pegunigalsidase alfa (**Figure 6**). For all samples except for those from patient 1, the rescued agalsidase alfa activities were lower than those for pegunigalsidase alfa, confirming our previous data demonstrating a lower affinity of pre-existing ADAs against pegunigalsidase alfa (20). The alkaline pretreated serum samples of patient 1 showed again only a slight inhibition of agalsidase alfa, potentially indicating that at the time-point of blood drawing a low titer of circulating ADAs might be present (**Figure 6**). To further address this observation and distinguish between anti-AGAL and anti-PEG antibodies, as wells as different antibody isotypes, we performed ELISAs against pegunigalsidase alfa, agalsidase alfa as well as PEG moieties with treated serum samples from the patients.

### 3.4 ELISA-based antibody characterization

**Figure 7:**
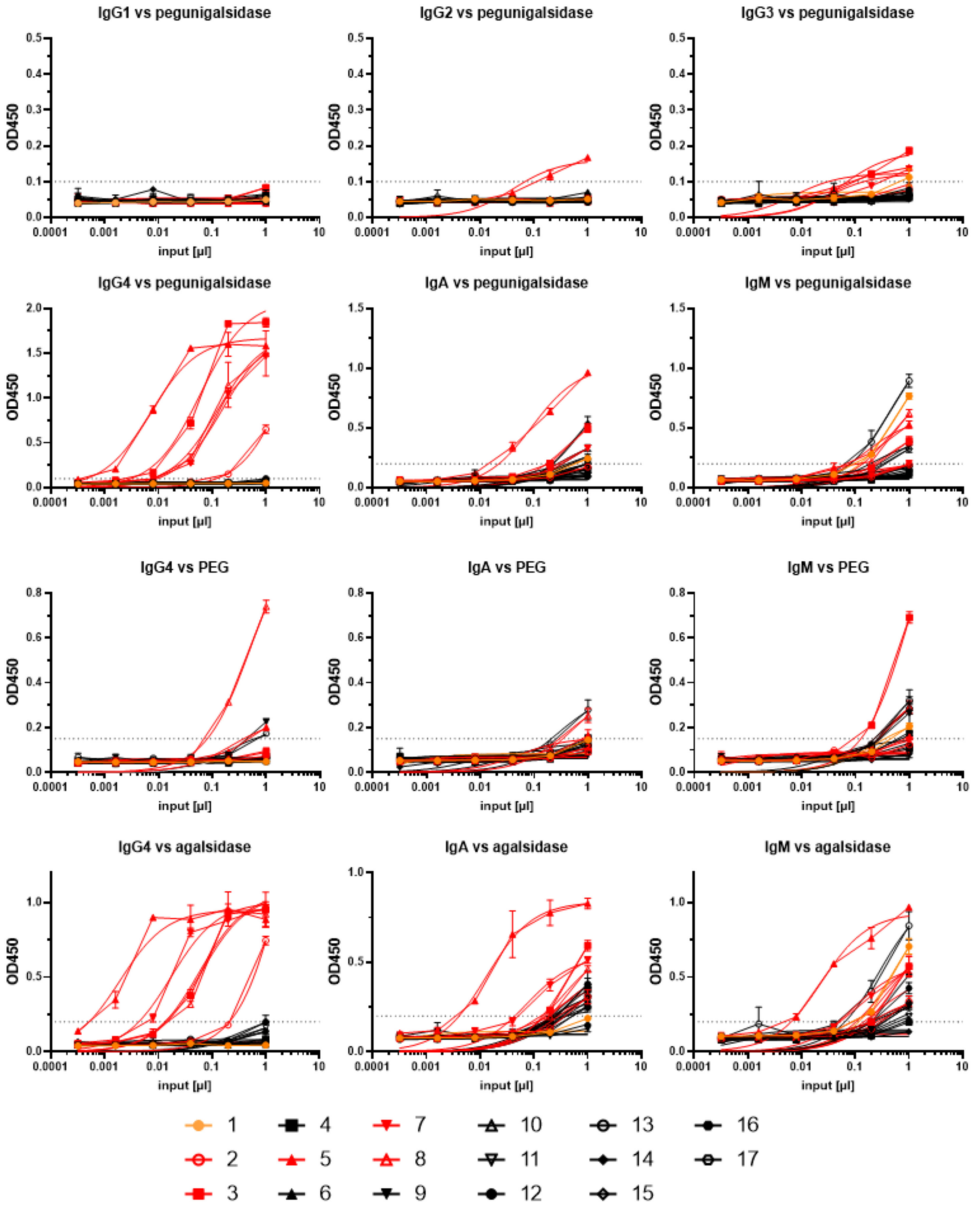
ELISA-based immunoglobulin isotyping in alkaline-pretreated serum samples from the last available visit from pegunigalsidase alfa-treated patients. ELISAs were performed against pegunigalsidase alfa, PEG and agalsidase alfa to distinguish between antibodies recognizing amino acid sequences or PEG moieties. The dotted lines mark the cut-off values for positive signals. Red lines indicate values in patients with pre-existing neutralizing antidrug antibodies. Black lines indicate values in patients without pre-existing neutralizing antidrug antibodies. Orange lines highlight values in patient 1.

Since neutralizing ADAs in FD against ERT are reported to belong mainly to IgG4 and also IgG1, we performed an isotyping including IgG1, IgG2, IgG3 and IgG4, but also for IgM and IgA for all serum samples from the last assessed infusion (either visit 3 or 4) (**Figure 7**). Only serum samples from patients with pre-existing neutralizing ADAs were positive for IgG4 against pegunigalsidase alfa (**Figure 7**). By contrast, ELISAs against IgM and IgA showed a more heterogenous distribution, demonstrating that 10 (58.8%) patients (patients 1, 2, 3, 5, 7, 8, 9, 13, 16, 17) were positive for IgM and 10 (58.8%) patients (patients 1, 3, 5, 6, 7, 8, 9, 13, 14, 16) were positive for IgA antibodies against pegunigalsidase alfa (**Figure 7**, **Supplementary Table 1**). To assess if the antibodies’ epitopes were directed against the amino acid backbone of pegunigalsidase alfa or against PEG moieties, ELISAs were repeated with all positive tested samples against PEG and agalsidase alfa (**Figure 7**, **Supplementary Table 1**). Only serum from patient 8 was tested high positive for an anti-PEG IgG4 antibody signal, while sera from patients 5, 9 and 13 showed only slight signals (**Figure 7**, **Supplementary Table 1**). Interestingly, sera from 7 patients (patients 1, 3, 8, 9, 13, 15, 17), were positive for IgM antibodies against PEG (**Supplementary Table 1**). Measurable IgA signals against PEG were only measured in patients 8 and 17 (**Figure 7**, **Supplementary Table 1**). As expected, patients with pre-existing ADAs were positive for IgG4, IgM and IgA antibodies against agalsidase alfa (**Figure 7**, **Supplementary Table 1**). While none of the patients without pre-existing neutralizing ADAs (including patient 1) were positive for IgG4 antibodies against agalsidase alfa, most patients were at least slightly positive for IgM and/or IgA antibodies against agalsidase alfa, too (**Figure 7**, **Supplementary Table 1**). Of note, one of the patients with the highest IgM antibody signal against agalsidase alfa was patient 1 (**Figure 7**).

## 4 Discussion

Neutralizing ADAs can limit the treatment efficacy of ERT in affected patients significantly. Therefore, regular measurement of antibody titers, in addition to recording clinical parameters, is an important aspect of assessing a patient's disease progression. Pegunigalsidase alfa is a newly approved promising ERT for the treatment of FD. Due to PEGylation, a half-life of 80 hours can be achieved, resulting in a measurable enzymatic AGAL activity detectable in the treated patient until the next infusion. However, this long half-life is an interfering factor when measuring any antibodies against the therapy. The aim of this work was to establish a protocol suppressing the pegunigalsidase alfa-mediated drug interference in standard assays used to measure free antibodies in patients at risk. In short, our main results are as follows: (1) by using an alkaline pretreatment of serum samples with subsequent neutralization, we established a simple and rapid protocol to overcome the pegunigalsidase alfa-mediated drug interference in antibody measurements; (2) the successful validation of this protocol allowed the application of serum-mediated inhibition assays in clinical practice and (3) a comprehensive immunoglobulin isotyping in pegunigalsidase alfa-treated patients; (4) one patient was identified with *de novo* non-inhibitory anti-pegunigalsidase alfa antibodies potentially leading to decreased pharmacokinetics by a premature elimination of pegunigalsidase alfa mediated by PBMCs.

### 4.1 Establishment of a simple and rapid protocol for the removal of drug interferences for antibody measurements

Standard techniques including serum-mediated inhibition assays and ELISA-based approaches for the measurement of anti-drug antibodies are based on the detection of free and thus unbound antibodies in patients’ sera. In comparison to the treatment with agalsidase alfa or beta, pegunigalsidase alfa and also not (yet) approved gene therapies can interfere with these assays due to the prolonged plasma half-life or the continuous AGAL expression. Thus, a pretreatment of (serum) samples is required, to eliminate these drug interferences. One already published method is based on the Melon gel-mediated purification of IgGs, a protocol which has been successfully implemented for the determination of the inhibitory capacity of neutralizing anti-drug antibodies against different ERTs in FD (9, 19). However, since this method is time and cost-intensive, a more feasible protocol for antibody screening is warranted. In this respect, our data demonstrate the successful denaturation of AGAL by a short incubation of sera with alkaline NaOH buffer. The use of a pH of 12.2 for AGAL inactivation and a subsequent neutralization was not to harsh to denaturate the free antibodies in respective serum samples. Furthermore, the use of NaOH and NaAc buffer, which ions are also used in enzymatic activity assays, enables us to measure the samples directly without an additional re-buffering or dialysis step, simplifying downstream applications. Our subsequent evaluation in serum samples drawn during and after infusions with agalsidase beta demonstrate that the chosen experimental setup and buffer conditions are capable to suppress the most *in vivo* AGAL concentrations. As previously highlighted (17, 18) the long half-life of pegunigalsidase alfa might lead to the formation of ADA/AGAL complexes in sera and thus to false negative measurements of antibody titers. As also seen for the samples from the agalsidase beta-receiving control patient with neutralizing ADAs, our protocol is not able to break down ADA/AGAL complexes, which is a limitation. Ideally, a breakdown of these complexes would result in further release of antibodies, which would be measurable in increased inhibition and thus decreased rescued AGAL activities. One reason why we were unable to detect complex degradation could be that the complexes either did not dissociate or that the antibodies bind again to AGAL after neutralization, meaning that no increase in titer was measurable. However, the latter can be largely ruled out, as at least the AGAL activity-neutralizing IgG4 antibodies no longer recognized the pretreated and neutralized pegunigalsidase alfa (as demonstrated in control ELISAs). It should be noted that higher NaOH concentrations and lower acidic pH values in our experiments led to irreversible denaturation of all antibodies (data not shown). However, the Western blots with serum samples from patients receiving pegunigalsidase alfa may provide a simpler explanation. Since no AGAL signals were detected in serum samples from patients with pre-existing antibodies taken immediately before the next infusions, it may be that no ADA/AGAL complexes were present at that time.

At least, free antibodies can lead to the *in-situ* formation of immune complexes in the case of subepithelial deposition of ERT enzymes (exogenous antigens) within the glomerulus and subsequent complement-cascade activation, which can result in membranous glomerulonephritis (5, 21). The clinical significance and impact of the complement activation of preformed circulating AGAL/ADA immune complexes in FD is unclear.

### 4.2 *In vivo* analysis of the pharmacokinetic profile of pegunigalsidase alfa after infusion

For further validation, we analyzed serum samples from 17 patients newly treated with pegunigalsidase alfa, demonstrating a comparable pharmacokinetic profile for pegunigalsidase alfa as previously described (14). Interestingly our Western blot analyses confirmed the complete physical absence of pegunigalsidase alfa in sera from patients with pre-existing antibodies. This indicates that the antibodies not only inhibit pegunigalsidase alfa, but seem to lead to a premature elimination from blood. An *in vivo* elimination of pegunigalsidase alfa by PBMCs could be demonstrated for patient 1, which confirms previous *in vitro* observations for agalsidase beta by Linthorst and colleagues (7). However, it is not entirely clear why this was not observed in patient 2 with pre-existing ADAs. It is possible that the rate of elimination is determined by the antibody titer and the composition of the immunoglobulin isotypes and their epitopes. Further analyses including a larger number of patients are now warranted to investigate this topic in more detail.

### 4.3 Antibody detection using serum-mediated AGAL inhibition assays and ELISA-based antibody characterization

Effect-based measurements such as serum-mediated inhibition tests are best suited for testing FD patients for ADAs, as they provide direct evidence of a therapy-neutralizing effect of anti-drug antibodies (7, 8). Our data show that alkaline pretreatment of serum samples can suppress the infused pegunigalsidase alfa and that the samples can be used for classic serum-mediated inhibition assays for the general detection of neutralizing ADAs. While noticeable inhibition was detected in the sera of all patients with pre-existing ADAs, only slight inhibition of pegunigalsidase alfa was detected in the serum of patient 1, although no pegunigalsidase alfa could be detected in the same serum. Since the patient did not have any neutralizing antibodies against AGAL previously, there could be several reasons for the low inhibition. First, since these were formed *de novo*, the measurable titer of free antibodies may still be too low. Furthermore, it is conceivable that these are mainly not inhibitory IgG4 antibodies, but rather other isotypes primarily mediating the elimination of pegunigalsidase alfa for example via PBMCs. Our comprehensive ELISA-based isotyping confirmed the latter hypothesis, since patient 1 was (yet) negative for IgG4, but positive for IgM antibodies, which are the first to be formed in a corresponding immune response. Interestingly, although female patient 13 showed a comparable immunoglobulin panel, we observed no significant effects on the pharmacokinetic profile of pegunigalsidase alfa in this patient. However, at least a slight premature elimination of the enzyme by PBMCs in this patient cannot be excluded due to absent samples.

Further long-term studies in patients are now warranted to investigate the effect of different antibody classes on the pharmacokinetics of pegunigalsidase alfa. In addition to serum-mediated inhibition assays, this includes isotyping of the antibodies and, ideally, serum detection of the enzyme and appropriate analyses in patients’ PBMCs.

There was a high prevalence of IgA and IgM signals against AGAL and PEG in our cohort. The origin of these signals, particularly in females is currently unknown. While the formation of anti-PEG antibodies could be triggered by cosmetics, processed foods, oral pegylated drugs, and pegylated mRNA-based vaccines i.m., the presence of non-inhibitory IgA and IgM antibodies against the amino acid sequence of AGAL cannot currently be explained. The effects of these non-inhibitory antibodies against the amino acid sequence of AGAL and against PEG are currently unclear and require further investigation. However, it is conceivable that high anti-PEG titers in particular could reduce the tolerability of pegunigalsidase alfa and thus the safety of the patient. Since agalsidase alfa contains polysorbate as an additive, which can cause a cross-reaction with anti-PEG antibodies, the tolerability of agalsidase alfa (cross-reaction) may also be limited if anti-PEG antibodies are detected.

## 5 Conclusion

We here present a simple and rapid alkaline treatment-based method to overcome drug interferences to measure at least free antibodies in patients treated with pegunigalsidase alfa. In combination with AGAL activity measurements and further biochemical characterization we were able to detect and determine the impact of *de novo* antibodies against pegunigalsidase alfa. Due to the increasing complexity resulting from the long half-life of pegunigalsidase alfa, targeted approaches tailored to the individual patient can be necessary to assess the impact of ADAs. Since ADAs can significantly limit the effectiveness of ERT, valid measurement of ADAs (without confounding factors) is of considerable clinical relevance. The use of our established assay can significantly improve the clinician's assessment of the course of the disease and the treatment decision for the patient.

## 6 Limitations

The aim of this study was the development of a rapid and feasible protocol to overcome the drug interference of pegunigalsidase alfa in antibody measurements. Although this has been achieved, the inability to break down any antibody-antigen complexes is a limitation. Therefore, the measured antibody titers and inhibitory capacities of the free antibodies can only be considered and interpreted as approximate values. Further studies, particularly with a larger number of patients, are needed to further characterize and confirm the potential effects of immunoglobulins and their isotypes on the activity of pegunigalsidase alfa and the potential elimination during and after infusion.

## Data Availability

The original contributions presented in the study are included in the article/**Supplementary Material**. Further inquiries can be directed to the corresponding author.
