# Supplementary material for: A rapid method to reduce drug interferences for antibody measurements in pegunigalsidase alfa-treated patients with Fabry disease
Source: Front Immunol. 2026 Jan 13;16:1724835. doi: 10.3389/fimmu.2025.1724835 (PMC12835379; doi:10.3389/fimmu.2025.1724835)
Supplement: Supplementary file 1 [file Table1.docx]

Supplementary Material

| **Supplementary Table 1: ELISA-based antibody detections from the most recent visits in pegunigalsidase alfa-treated patients.** | | | | | | | | | |
| --- | --- | --- | --- | --- | --- | --- | --- | --- | --- |
|  | **anti-pegunigalsidase antibodies** | | | **anti-PEG antibodies** | | | **anti-agalsidase alfa antibodies** | | |
| **patient** | **IgG1/2/3/4** | **IgA** | **IgM** | **IgG4** | **IgA** | **IgM** | **IgG4** | **IgA** | **IgM** |
| 1 | -/-/-/- | + | + | - | (+) | + | - | - | + |
| 2 | -/-/-/+ | - | + | - | - | - | + | + | + |
| 3 | -/-/(+)/+ | + | + | - | - | + | + | + | + |
| 4 | -/-/-/- | - | - | - | (+) | - | - | (+) | (+) |
| 5 | -/(+)/(+)/+ | + | + | (+) | (+) | (+) | + | + | + |
| 6 | -/-/-/- | + | - | - | - | - | - | (+) | (+) |
| 7 | -/-/(+)/+ | + | + | - | - | - | + | + | + |
| 8 | -/-/-/+ | + | + | + | + | + | + | + | + |
| 9 | -/-/-/- | + | + | (+) | - | + | - | - | + |
| 10 | -/-/-/- | - | - | - | - | - | - | (+) | - |
| 11 | -/-/-/- | - | - | - | - | - | - | (+) | - |
| 12 | -/-/-/- | - | - | - | - | (+) | (+) | (+) | + |
| 13 | -/-/-/- | + | + | (+) | (+) | + | - | + | + |
| 14 | -/-/-/- | + | - | - | - | - | - | - | - |
| 15 | -/-/-/- | - | - | - | - | + | - | (+) | (+) |
| 16 | -/-/-/- | (+) | (+) | - | (+) | (+) | - | (+) | + |
| 17 | -/-/-/- | - | + | - | + | + | - | + | + |
| -: negative for corresponding antibodies. (+): slightly positive for corresponding antibodies, +: positive for corresponding antibodies. | | | | | | | | | |
|  | | | | | | | | | |

| 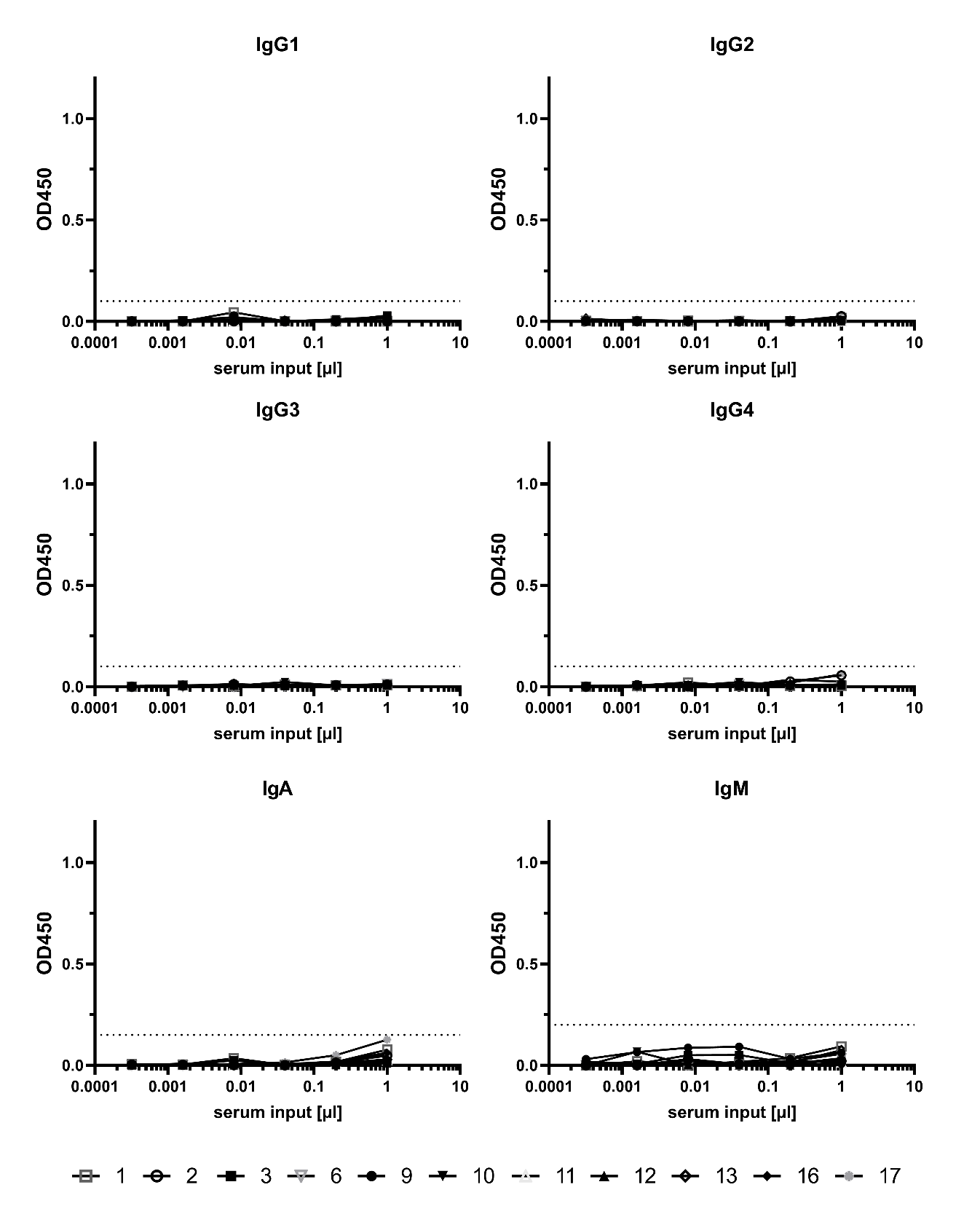 |
| --- |
| **Supplementary Figure 1: Control ELISAs with alkaline-pretreated serum samples against BSA demonstrating no markable unspecific binding of analyzed Igs.** The dotted lines mark the cut-off values for positive signals. |

| 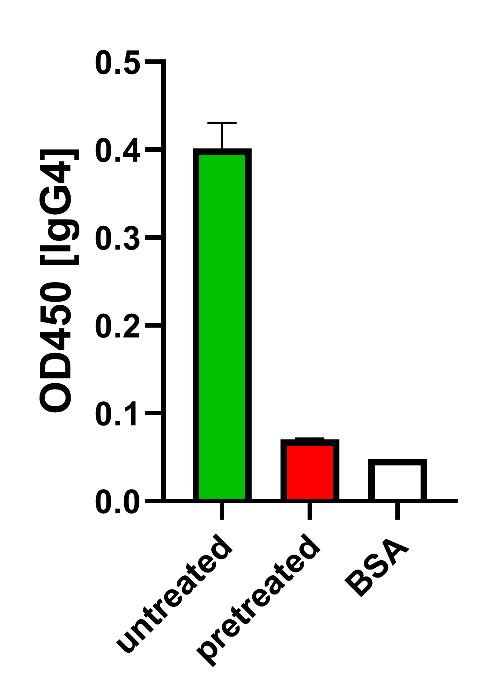 |
| --- |
| **Supplementary Figure 2: Control ELISA against pegunigalsidase alfa.** Wells were either coated with untreated or pretreated and neutralized pegunigalsidase alfa or BSA as control. The patient derived reference anti-AGAL IgG antibody showed a significantly reduced affinity against pretreated and thus eliminated pegunigalsidase alfa. A reassembly of dissociated AGAL/ADA complexes after neutralization can therefore be excluded. |

| 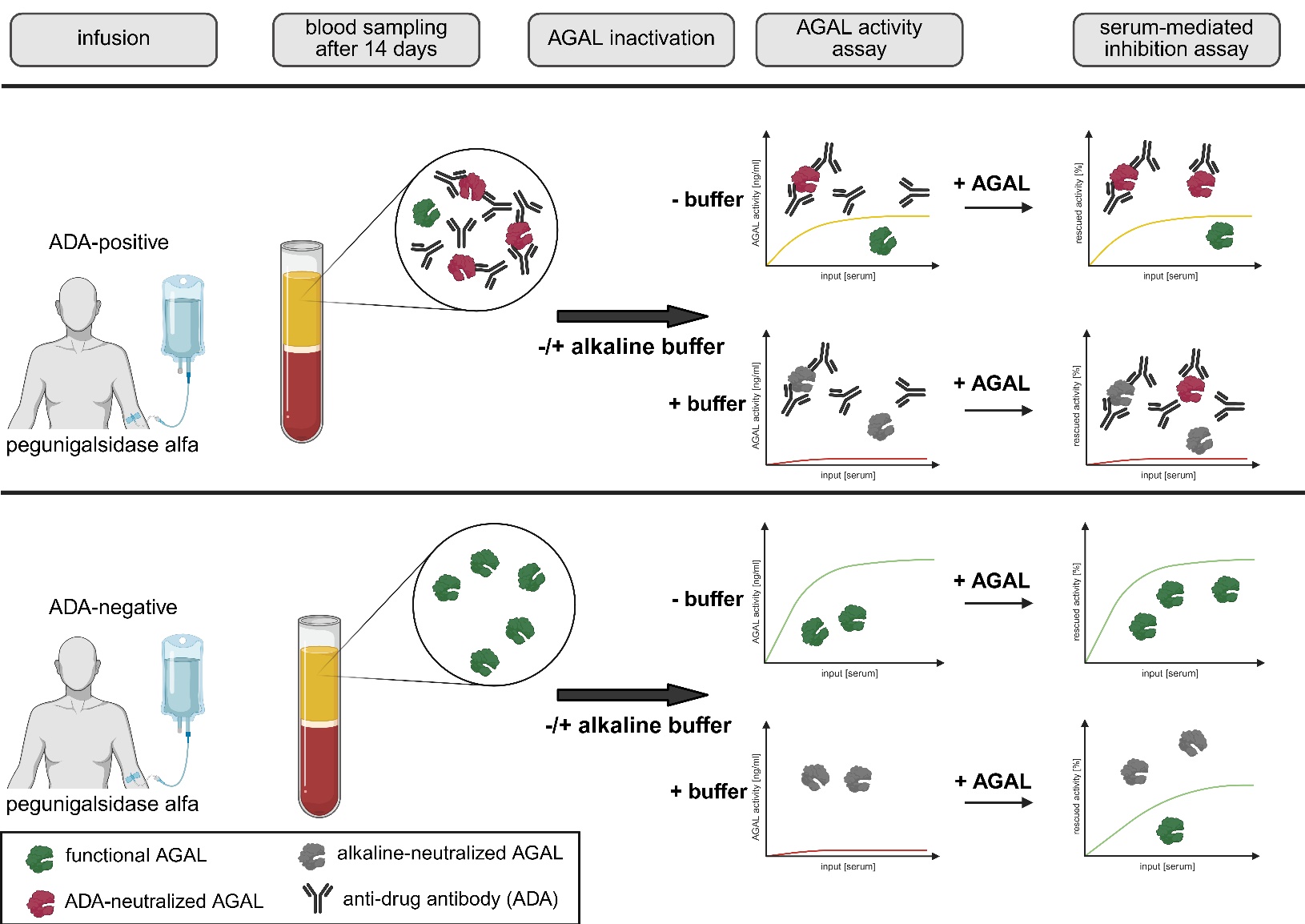 |
| --- |
| **Supplementary figure 3: Setup and background for the conduction of the serum-mediated inhibition assays with pretreated serum samples to detect inhibitory antibodies against pegunigalsidase alfa.** |

| 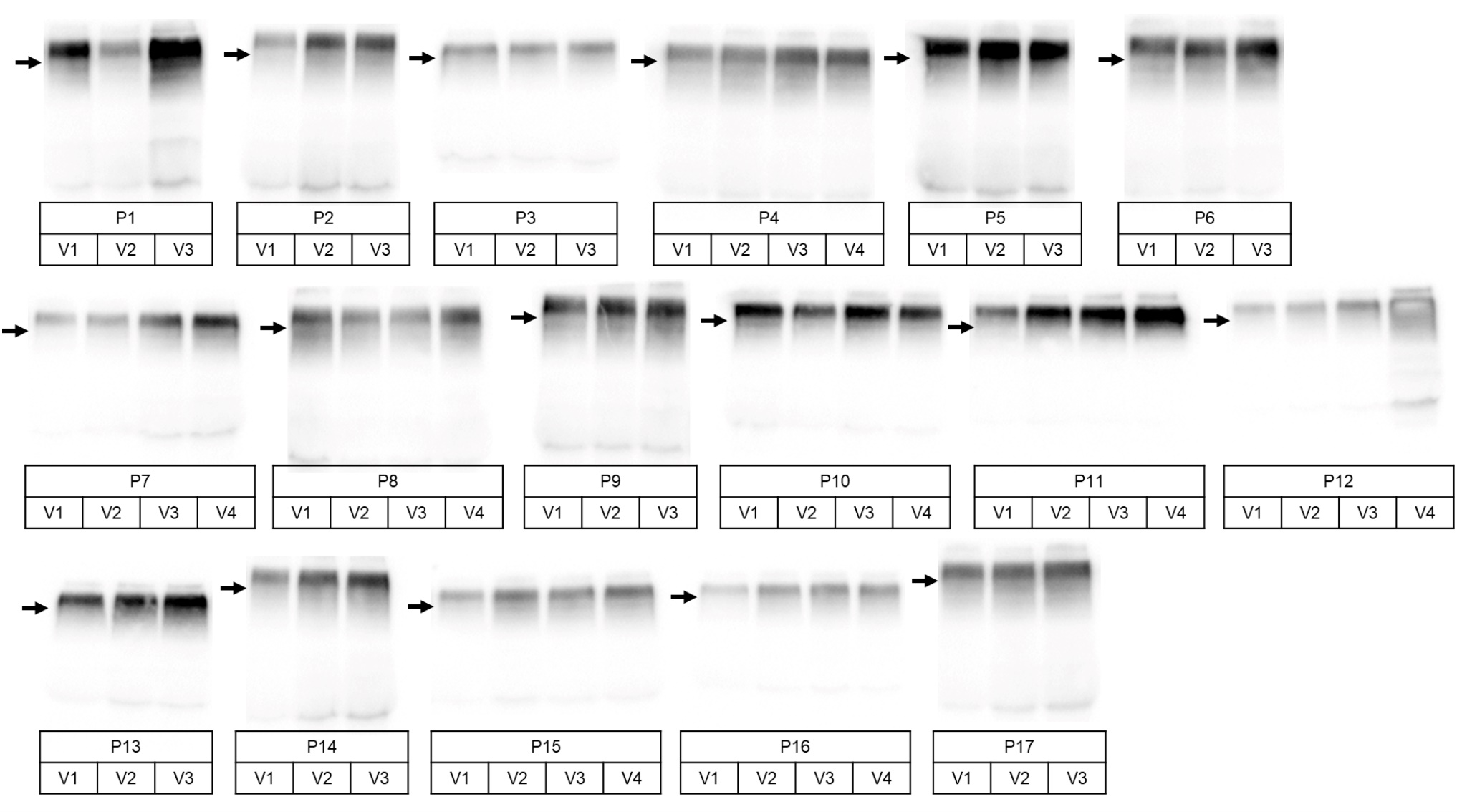 |
| --- |
| **Supplementary Figure 4: Non-quantitative Western Blots with serum samples drawn directly after infusions for individual pegunigalsidase alfa detection.** The arrows indicate the running height of pegunigalsidase alfa. Since the serum samples were immobilized on individual membranes, no comparisons between patients can be made. |
